# Distribution and diversity of anaerobic thermophiles and putative anaerobic nickel-dependent carbon monoxide-oxidizing thermophiles in mesothermal soils and sediments

**DOI:** 10.3389/fmicb.2022.1096186

**Published:** 2023-01-09

**Authors:** Amber N. DePoy, Gary M. King

**Affiliations:** Department of Biological Sciences, Louisiana State University, Baton Rouge, LA, United States

**Keywords:** thermophile, mesophile, anaerobic, carbon monoxide, soil, sediment, microbial diversity, biogeography

## Abstract

Even though thermophiles are best known from geothermal and other heated systems, numerous studies have demonstrated that they occur ubiquitously in mesothermal and permanently cold soils and sediments. Cultivation based studies of the latter have revealed that the thermophiles within them are mostly spore-forming members of the Firmicutes. Since the geographic distribution of spores is presumably unconstrained by transport through the atmosphere, similar communities (composition and diversity) of thermophiles might be expected to emerge in mesothermal habitats after they are heated. Alternatively, thermophiles might experience environmental selection before or after heating leading to divergent communities. After demonstrating the ubiquity of anaerobic thermophiles and CO uptake in a variety of mesothermal habitats and two hot springs, we used high throughput sequencing of 16S rRNA genes to assess the composition and diversity of populations that emerged after incubation at 60°C with or without headspace CO concentrations of 25%. Anaerobic Firmicutes dominated relative abundances at most sites but anaerobic thermophilic members of the Acidobacteria and Proteobacteria were also common. Nonetheless, compositions at the amplicon sequence variant (ASV) level varied among the sites with no convergence resulting from heating or CO addition as indicated by beta diversity analyses. The distinctions among thermophilic communities paralleled patterns observed for unheated “time zero” mesothermal soils and sediments. Occupancy analyses showed that the number of ASVs occupying each of *n* sites decreased unimodally with increasing *n*; no ASV occupied all 14 sites and only one each occupied 11 and 12 sites, while 69.3% of 1873 ASVs occupied just one site. Nonetheless, considerations of distances among the sites occupied by individual ASVs along with details of their distributions indicated that taxa were not dispersal limited but rather were constrained by environmental selection. This conclusion was supported by βMNTD and βNTI analyses, which showed dispersal limitation was only a minor contributor to taxon distributions.

## Introduction

Environments that support active thermophilic bacterial growth are widespread but represent a very small fraction of the Earth’s surface. Nonetheless, thermophiles are essentially ubiquitous, with numerous isolates derived from temperate and cold systems, including the phyllosphere, sludges, Arctic and Antarctic soils as well as permanently cold deep-sea sediments (Marchant et al., 2002; Hernon et al., 2006; Wu et al., 2006; Logan and Allan, 2008; Müller et al., 2014; Godon et al., 2020). Many of these isolates belong to the aerobic spore-forming Firmicutes, although non-sporing isolates have also been reported (Marchant et al., 2002; Logan and Allan, 2008). The presence of spore formers, sometimes at anomalously high concentrations, has led to speculation that many thermophiles might be inactive *in situ* and merely maintained by continuous inputs from geothermally heated sources (Marchant et al., 2008; Zeigler, 2014).

Taking the essentially ubiquitous distribution of *Geobacillus* (many of which have been reclassified as *Parageobacillus* [Najar and Thakur, 2020]) as an example, Zeigler (2014) has proposed that global scale dispersal through the atmosphere of spores that accumulate passively in cool soils and sediments accounts for the paradox of widely distributed, relatively abundant aerobic thermophiles in a largely temperate world. An analogous proposal has been presented for anaerobic thermophiles in deep-sea sediments, with ocean currents accounting for the dispersal of spores presumably originating from hydrothermal vents or other heated systems, e.g., Guyamas Basin (Müller et al., 2014). Although these proposals have focused on Firmicutes, they might apply more generally to a wide range of thermophiles, since the troposphere and the oceans represent well known media for short-and long-range transport of phylogenetically diverse taxa originating from numerous sources (e.g., Smith et al., 2013; Schmale and Ross, 2015; Maki et al., 2019; Santl-Temkiv et al., 2022).

If the distribution of terrestrial and marine thermophiles is not limited by dispersal through the atmosphere or by ocean currents (e.g., Barberán et al., 2014; Louca, 2022), then compositionally similar communities of each might be expected in geographically distant terrestrial and marine habitats, assuming that little or no environmental selection occurs post-deposition. This proposition has been partially tested by a study of marine anaerobic thermophilic Firmicutes (with “anaerobic thermophilic” simplified as “antherm,” e.g., antherm-Firmicutes). Contrary to the proposition, results from Müller et al. (2014) indicated that communities that developed after incubation at 50°C differed spatially, with none of the observed taxa universally distributed; instead, some populations were described as cosmopolitan while others were apparently limited by circulation patterns.

Müller et al. (2014) proposed that dispersal limitation accounted for the results and discounted environmental selection as a factor in the community differences they observed, because water column and sediment temperatures were never within ranges permissive for thermophile activity. However, this might not be the case for terrestrial systems, where temperatures can occasionally include permissive ranges even in typically cold habitats (Cockell et al., 2014; Santana and Gonzalez, 2015). Differences in thermophile community compositions among terrestrial systems could therefore arise from selective losses of some taxa and occasional growth of others (Cockell et al., 2014; Wilson and King, 2022). While evidence to support these notions has been provided by culture-based studies, there have been few tests of the concepts based on observations of intact soils under *in situ* or *ex situ* conditions.

Indeed, most of what is known about thermophilic populations and communities in temperate soils has been derived from culture-based studies (e.g., McMullan et al., 2004) with only a few cultivation-independent studies reported to date. Norris et al. (2002) used denaturing gradient gel electrophoresis (DGGE), 16S rRNA gene clone libraries, and cultivation methods to assess microbial community responses to a rapid geothermal heating event in a lodgepole pine stand at Yellowstone National Park (USA). Results from soils obtained along a thermal gradient approximately 3 months after heating had been initiated and from soils incubated at 50°C in a 4-week microcosm experiment revealed both non-sporing and spore-forming thermophiles in the seed banks of unheated soils.

A later study of mesothermal volcanic soils incubated anaerobically at 60°C revealed diverse communities of Firmicutes-dominated thermophiles at each of three variably vegetated sites with significant contributions of non-sporing Proteobacteria and other phyla in an 800-year old forested site (DePoy et al., 2020). The outcomes indicated that site age and plant development contributed to anaerobic thermophilic community composition and suggested that the assembly of thermophilic communities in mesothermal habitats is more complex than Zeigler (2014) proposed, likely involving a variety of global and local processes.

In a subsequent study, DePoy and King (2022) assessed the geographic distribution and potential activity of putative nickel-dependent anaerobic carbon monoxide-oxidizing bacteria (Ni-COX) under anaerobic mesophilic and thermophilic conditions. That study was conducted to compare CO uptake capacities of a wide range of soils and sediments under oxic and anoxic conditions with environmentally relevant CO concentrations (10 ppm). In addition, a set of analyses was conducted under anoxic conditions with 25% CO to assess potential activities attributable specifically to Ni-COX, which have been largely recognized as thermophiles though some mesophiles are known Fukuyama et al. (2020). A sub-set of the sites in that study were subjected to high throughput 16S rRNA gene sequence analyses to determine the composition and diversity of anaerobic thermophiles, including those that responded to CO additions. The results were used to evaluate the extent to which the composition and structure of thermophilic communities that emerged after heating converged, as anticipated if temperature was the primary structuring variable, or diverged reflecting important roles for other variables, e.g., site-based selection. The outcomes indicated that site-based selection was a major factor in community assembly while dispersal limitation played only a minor role. The results also indicated that even though anaerobic spore-forming Firmicutes dominated relative abundances at most sites, anaerobic thermophilic Acidobacteria and Proteobacteria as well as non-spore forming Firmicutes were also abundant, suggesting that transport models for thermophiles must be extended beyond spore-formers.

## Materials and methods

### Site descriptions

Samples for analyses of microbial community responses to elevated temperature and 25% CO were collected from Hawai’i, Oregon, and Louisiana (USA) and from Iceland and Japan. These sites represent a subset of those described by DePoy and King (2022), some of which have been described previously (King et al., 2008; Guo et al., 2014; Fujimura et al., 2016; DePoy et al., 2020). Briefly, Hawaiian sites included a forested site on Mauna Loa at Kipukakulalio (KKL) dominated by a stand of *Acacia koa* with an understory of grasses, an additional site at Kipukakulalio that had hosted *A*. *koa*, but that was impacted by a forest fire (KKL-burned) and surface sediment (upper 5–10 cm depth) from the shore of Lake Waiau (LWH) on Mauna Kea. Sites from Oregon included a managed, irrigated agricultural soil supporting *Medicago sativa* (Kueny Ranch Cultivated, KRC), and sediment (upper 5 cm) from mesothermal and heated sites at two hot springs (Mickey Hot Springs [MHS] and Alvord Hot Springs [AHS]; DePoy and King, 2022). Sediment was also obtained from a forested wetland in Louisiana (upper 5–10 cm, Bluebonnet Swamp, BBS). Samples from Iceland were obtained from the soil crust that had developed on an ~300-year old lava flow at the Krafla Geothermal Field (KRF). Samples from Japan were obtained from an experimental rice paddy on the campus of Ibaraki University (Ibaraki Rice, IJR) and from three sites on Miyake-jima (a forested site, CL; a grass-dominated site on a recent volcanic deposit, IG-7; a sparely vegetated site on a recent volcanic deposit, OY). The latter have been previously described by Guo et al. (2014), Fujimura et al. (2016) and DePoy et al. (2020). At each site, triplicate samples of approximately 100-gram fresh weight (gfw) were collected using ethanol-sterilized spatulas or trowels. Samples were transferred to zip-seal storage bags and maintained at ambient temperature during transport to a laboratory at Louisiana State University for further processing. Physical and chemical variables for all samples were analyzed as described by DePoy et al. (2020); data, including GPS coordinates, are available as Table 1 and Supplementary Table 1.

**Table 1:** Sites used in this study and their physical characteristics (temperature at 2 cm depth, °C; organic content, loss on ignition % of dry weight; values for pH and organic contents are means of triplicate determinations ±1 standard error).

Site name	Abbreviation	Sample type	Temperature	pH	Organic content
Kueny Ranch Cultivated	KRC	Agricultural soil	28.9	7.1 ± 0.08	6.8 ± 0.3
Kueny Ranch Cultivated	KRC-2	Agricultural soil	29.2	–	5.5 ± 0.2
Climax Forest Miyake-jima	CL	Forest soil	24.1	5.2 ± 0.05	21.8 ± 0.5
Kipukakulalio	KKL	Forest soil	16.8	5.5 ± 0.04	31 ± 1.2
Kipukakulalio-burned	KKL-burn	Forest soil	24.7	5.8 ± 0.06	42.9 ± 9.87
Krafla Geothermal Field	KRF	Volcanic soil	10.1	6.2 ± 0.06	16.3 ± 1.5
Krafla Geothermal Field	KRF-2	Volcanic soil	9.7	–	9.3 ± 0.4
Igaya, O-yama Volcano	IG-7	Volcanic soil	22.1	4.5 ± 0.03	1.7 ± 0.1
O-yama Volcano	OY	Volcanic soil	22.4	4.8 ± 0.05	3.1 ± 0.4
Alvord Hot Springs	AHS-30	Hot spring	30	8.0 ± 0.05	7.9 ± 0.4
Alvord Hot Springs	AHS-60	Hot spring	60	7.2 ± 0.32	9.5 ± 0.7
Alvord Hot Springs	AHS-70	Hot spring	74	7.5 ± 0.28	4.5 ± 0.3
Mickey Hot Springs	MHS-25	Hot spring	25.2	8.3 ± 0.12	10.2 ± 1
Mickey Hot Springs	MHS-60	Hot spring	60	9.0 ± 0.08	4.2 ± 0.4
Mickey Hot Springs	MHS-69	Hot spring	69.2	7.3 ± 0.03	4.9 ± 0.2
Bluebonnet Swamp	BBS	Flooded soil	18.2	5.8 ± 0.08	25.9 ± 1.1
Ibarki, Japan Rice	IJR	Flooded soil	13.1	5.2 ± 0.12	9.6 ± 0.3
Lake Waiau, Hawai’i	LWH	Sediment	6.5	7.2 ± 0.07	9.8 ± 4.4

Dashes indicate assays were not conducted. Additional details are available in the text and Supplementary Table 1.

### DNA extraction, sequencing, and analysis

As described by DePoy and King (2022), five-gfw from each of the triplicate samples at each site were transferred to 60-cm^3^ serum bottles that were sealed with blue butyl rubber stoppers. Anoxic headspaces were established by flushing the bottles with deoxygenated nitrogen. Two sets of triplicates from each site were incubated at 25°C, one with and one without 25% CO; two additional sets were incubated with and without 25% CO at 60°C. CO from a 100% stock was used to establish the CO treatments. CO concentrations were measured at intervals by collecting headspace samples with a needle and syringe for analysis by gas chromatography (DePoy et al., 2020). Briefly, headspaces were sampled (0.5 cm^3^) at intervals with a nitrogen-flushed needle and syringe for CO analysis using an SGI 8610C Gas Chromatograph (Folsom, CA, USA) equipped with a thermal conductivity detector and a Molecular Sieve 5A column (2 m × 6.25 mm OD stainless steel) operated at 60°C. CO uptake rates have been reported by DePoy and King (2022).

After CO was depleted, the bottles were opened and 1–2 gfw subsamples of the soil or sediment were collected with a spatula. The subsamples were stored at −80°C prior to extracting genomic DNA using a DNeasy PowerSoil extraction kit (Qiagen, source) following the manufacturer’s instructions. DNA extracts were visualized by gel electrophoresis and then shipped on dry ice to the Research Technology Support Facility at Michigan State University for multiplexed sequencing of the V4-V5 region of the 16S rRNA genes (primers 515F and 806R) with an Illumina Miseq platform with a 2 × 250 bp paired-end chemistry. Sequences from CL, IG-7 and OY were analyzed in a previous study (PRJNA673894) and combined with those from this study. The latter sequences have been deposited as PRJNA892052.

Sequences were processed as described by DePoy et al. (2020). Briefly, sequencing yielded a total of 19,309,368 reads. The DADA2 pipeline (Callahan et al., 2016) was used to generate amplicon sequence variants (ASVs), resulting in 13,340,277 reads that were classified using the SILVA v132 database (Quast et al., 2013). Analyses of diversity and composition and data visualizations were conducted using the phyloseq R package (McMurdie and Holmes, 2013). Prior to analysis, the data were filtered to remove samples with ≤2,500 reads; the remaining data were then split by incubation temperature (i.e., 25°C and 60°C samples were analyzed separately). ASVs for occupancy pattern assessments were constrained to those with greater than four counts. For all other analyses of samples incubated at 25°C, the most informative ASVs were selected based on abundance and variance (at least four counts in 5% of the samples). For the 60°C set, the ASVs were screened to identify candidate thermophilic anaerobes. Candidate thermophilic anaerobes were defined on the basis of literature searches and BLAST analyses to verify taxonomic assignments. Candidate thermophilic anaerobes included ASVs that were members of genera or higher taxonomic ranks known to harbor anaerobic thermophiles (e.g., *Anoxybacillus*, Aminicenantales, Thermodesulfovibrionia). All ASVs originally identified as *Geobacter* were subsequently assigned to *Parageobacillus* based on the outcomes of BLAST analyses. ASVs identified as mesophiles (e.g., *Bryobacter*) or thermophilic aerobes (e.g., *Conexibacter*) were excluded from further analyses of the 60°C dataset. Specific genera were designated as putative Ni-dependent CO oxidizers or putative Ni-COX on the basis of literature reports of CO oxidation or identification of the Ni-CODH genes in genome analyses.

For alpha diversity estimates (S_obs_ [richness] and Shannon Index [abundance and evenness]), data from the time zero (T_0_), 25°C, and 60°C incubations were normalized to the minimum sample size using the SRS package (Beule and Karlovsky, 2020). For beta diversity analyses, the data from the three sets were transformed using a total sum scaling followed by ordinations using weighted and unweighted UniFrac metrics (Lozupone and Knight, 2005). Trees used for UniFrac were constructed using FastTree (Price et al., 2010; Liu et al., 2011). Statistical analyses for beta diversity metrics were analyzed using adonis in the vegan R package (Dixon, 2003) and pairwise comparisons of the sites were conducted using the pairwiseAdonis R package (Martinez Arbizu, 2020). Dispersions for the replicates for each site were analyzed using betadisper in vegan (Dixon, 2003). Statistical analyses for the beta dispersions were performed using ANOVA followed by Tukey’s *Post Hoc* test in R. ASVs that showed differential abundances between the no CO and 25% CO treatments at each site were identified using DESeq2, with only ASVs with relative abundance >1% for at least one replicate considered (Love et al., 2014). The strength of ecological processes dominating community assembly were inferred using the beta nearest taxon index (betaNRI, or βNRI) and Bray-Curtis-based Raup-Crick (RCbray) RCBray using the microeco package (Stegen et al., 2013; Liu et al., 2021). Taxonomic barplots were prepared using the microshades R package (Dahl et al., 2022).

## Results

### Taxonomic composition of soils and sediments incubated at 60°C

**Figure 1:**
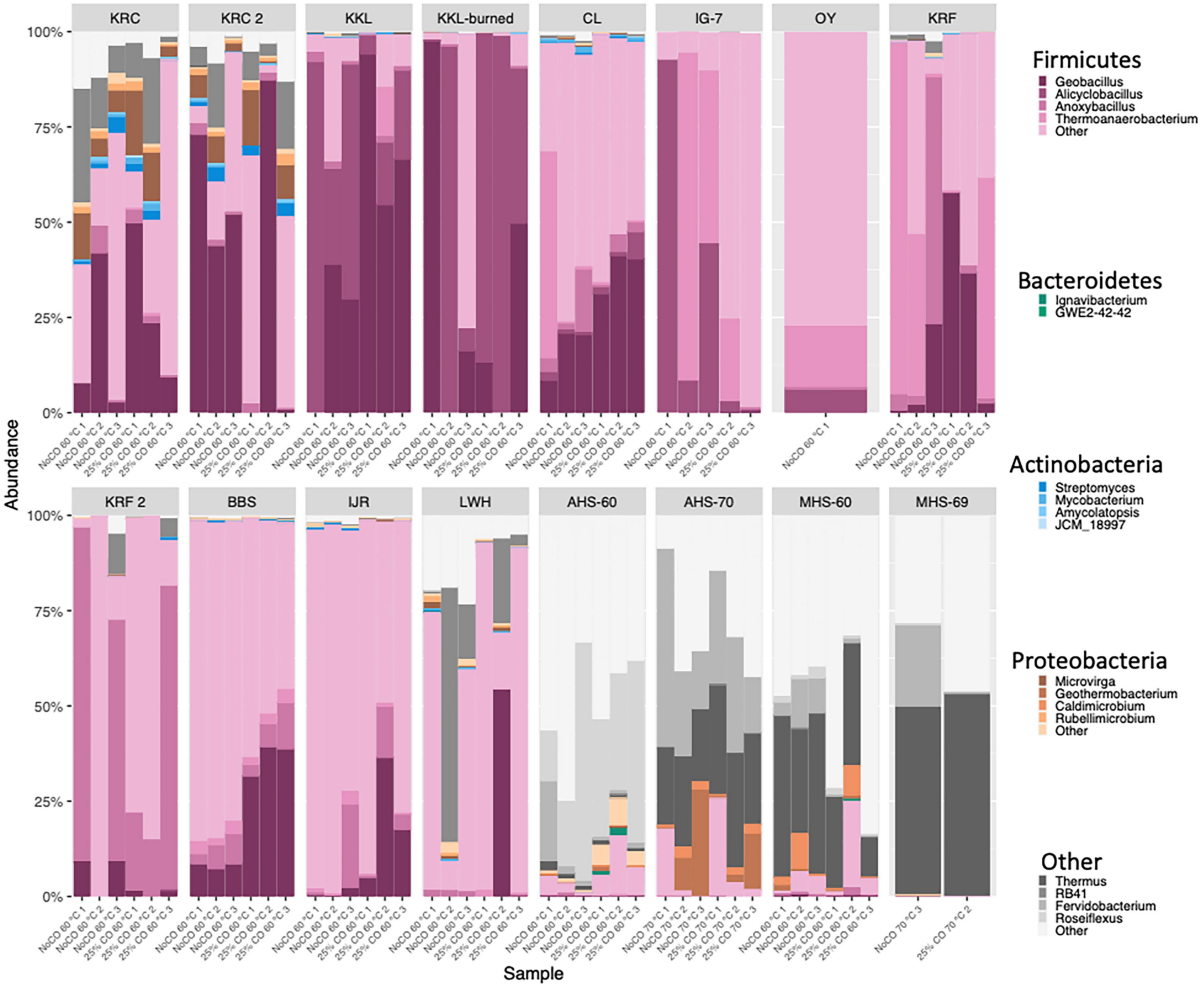
Taxonomic composition (phyla and genera indicated) for each of the sample replicates incubated under thermophilic conditions with or without 25% CO from which DNA was successfully extracted and amplified. KRC, Kueny Ranch Cultivated (1 = July, 2018; 2 = July, 2019); KKL, Kipukakulalio; KKL-burned, a stand of *Acacia koa* burned by a forest fire; CL, an 800-year old Miyake-jima forest; IG-7, vegetated 18-year old volcanic soils on O-yama Volcano (Miyake-jima); OY, sparsely vegetated 18-year old volcanic soils on O-yama Volcano; KRF, soil crust on an ~300-year old lava flow at Krafla Geothermal Field (1 = August, 2018; 2 = August, 2019); BBS, Bluebonnet Swamp, Louisiana; IJR, rice paddy, Ibaraki, Japan; LWH, Lake Waiau, Hawai’i; AHS, Alvord Hot Springs, Oregon (sample temperatures indicated by numbers), MHS, Mickey Hot Springs, Oregon (sample temperatures indicated by numbers).

Forested soils were dominated by candidate antherm-Firmicutes (86.0–99.9% of all reads); prominent genera included *Alicyclobacillus*, *Parageobacillus*, and *Thermoanaerobacterium*. Candidate antherm-Firmicutes also dominated volcanic soils (72.3–100% of all reads; Figure 1; Supplementary Table 2) but with a more diverse set of genera, including *Thermoanaerobacterium*, *Gelria*, *Moorella*, *Anoxybacillus*, *Tuberbacillus*, *Kyrpidia*, *Caldinitratiruptor*, *Caldanaerobius*, and *Parageobacillus*. Candidate antherm-Firmicutes (28.8–90.1% of all reads; Figure 1; Supplementary Table 2) and candidate antherm-Actinobacteria (3.28–31.9% of all reads; Figure 1; Supplementary Table 2) dominated a cultivated soil, with *Parageobacillus*, *Caldinitratiruptor*, and *Bacillus* (Firmicutes) and unclassified Solirubrobacterales (Actinobacteria) among the dominant groups. Hot springs were dominated by candidate antherm-Chloroflexi (1.98–55.8% of all reads; Figure 1; Supplementary Table 2) and Euryarchaeota (0–52.9% of all reads; Figure 1; Supplementary Table 2) with *Thermus*, *Fervidobacterium*, unclassified Anaerolineae, *Methanothermobacter*, and *Roseiflexus* as abundant genera. Candidate antherm-Firmicutes dominated flooded soils and sediments (20.9–99.1% of all reads; Figure 1; Supplementary Table 2) with a distinct set of prominent genera, which included *Carboxydothermus*, *Caloramator*, unclassified Solirubrobacterales, *Parageobacillus*, and *Fonticella*.

### CO responses by candidate thermophilic ASVs

**Figure 2:**
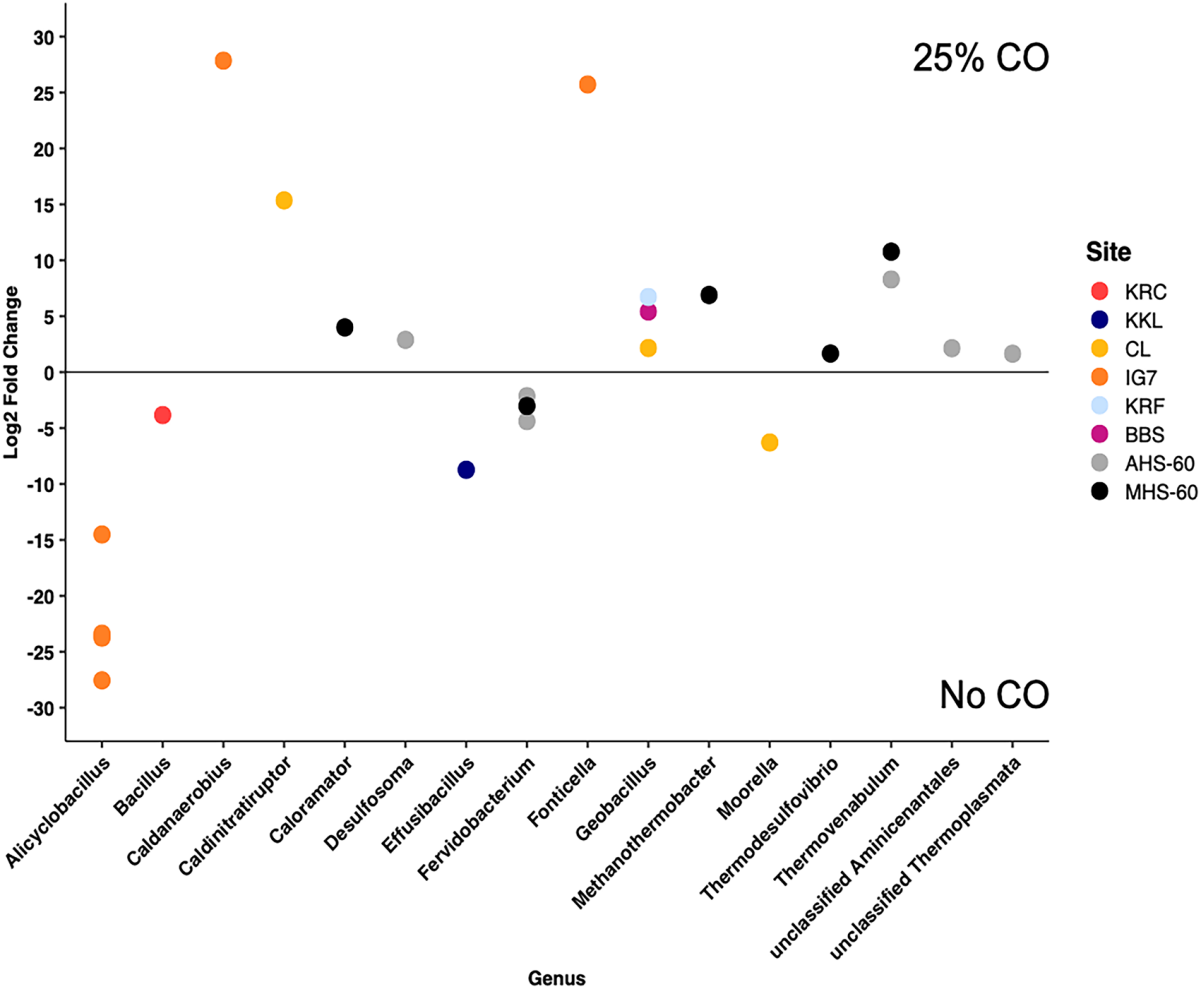
Differentially abundant ASVs identified in samples incubated at 60°C with or without 25% CO. Only significantly different ASVs (*p* ≤ 0.05) found in at least one replicate within a site and with relative abundances ≥1% are shown. The genus of each ASV is indicated. All points with positive Log2 Fold Changes are more abundant in the 25% CO treatment, all points with negative Log2 fold changes are more abundant in the No CO treatment. Site abbreviations are described in the legend for Figure 1.

Although CO addition did not significantly affect community composition, some ASVs differed between incubations with and without CO based on an analysis using DESeq2 of ASVs present at a relative abundance >1% in at least one replicate (Figure 2). Eleven ASVs differed for the two hot spring sites at 60°C (MHS-60 and AHS-60). Four of six ASVs for AHS-60 were more abundant with 25% CO, including the genera *Desulfosoma* (putative Ni-COX), an unclassified Aminicenantales, *Thermovenabulum,* and an unclassified Thermoplasmata. Two ASVs declined in abundance (both *Fervidobacterium*). Four of the five ASVs for MHS-60 were more abundant with 25% CO, including the genera *Thermovenabulum*, *Caloramator*, *Thermodesulfovibrio* (putative Ni-COX), and *Methanothermobacter* (putative Ni-COX). One ASV declined in abundance (*Fervidobacterium*). For a flooded soil site (BBS), one ASV (*Parageobacillus*, putative Ni-COX) was significantly more abundant with 25% CO. For the cultivated site (KRC and KRC-2), one ASV (*Bacillus*) declined in abundance for the CO treatment; no ASVs were enriched. At the forested CL site, two out of three ASVs that differed significantly were more abundant with 25% CO, including ASVs representing *Caldinitratiruptor* and *Parageobacillus* (putative Ni-COX); one ASV representing the genus *Moorella* (putative Ni-COX) was significantly more abundant for the no CO treatment. One ASV (*Effusibacillus*) for KKL was significantly more abundant for the no CO treatment.

### Candidate anaerobic thermophile alpha and beta diversity

**Figure 3:**
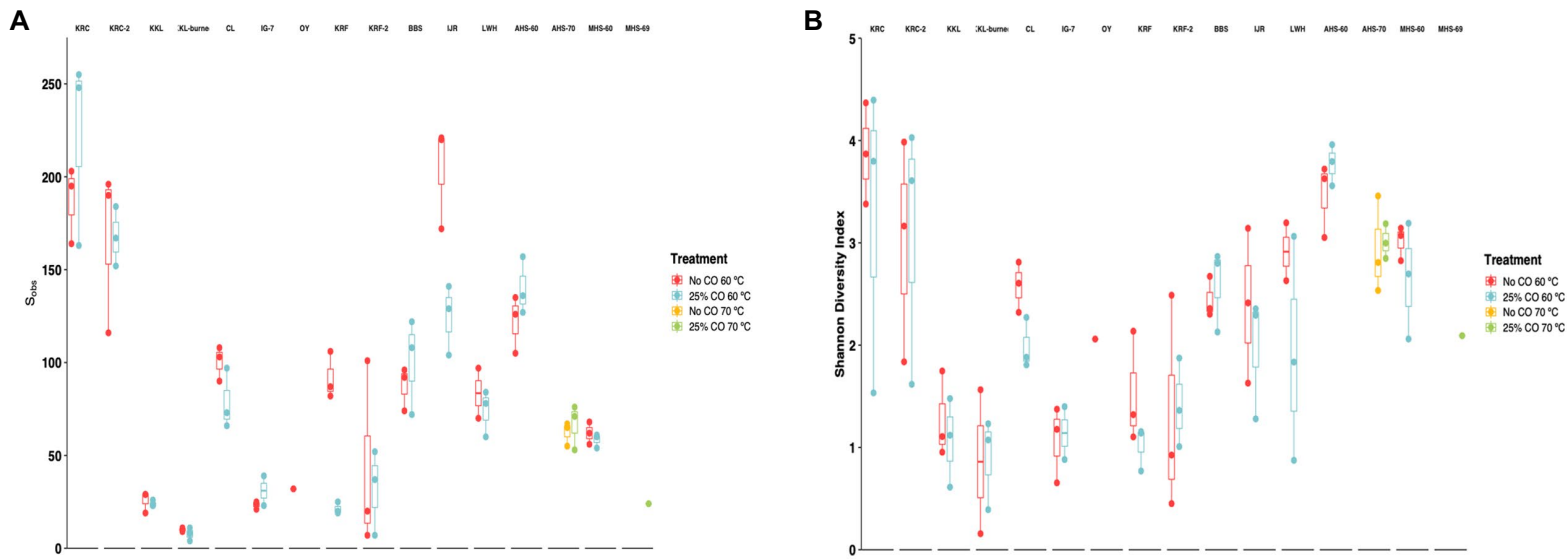
Alpha diversity for metrics for samples incubated under thermophilic conditions with and without 25% CO. Observed richness, Sobs **(A)** and Shannon diversity index **(B)** Bars in box and whisker plots represent median values. Site abbreviations are described in the legend for Figure 1.

For the 60°C dataset, samples were normalized to a minimum depth of 2,600 reads before estimating alpha diversity indices (S_obs_ and Shannon Index). S_obs_ estimates for species richness ranged from 7.67 ± 2.03 (KKL-burned, 25% CO, 60°C) to 222 ± 29.6 (KRC, 25% CO, 60°C) (Figure 3A; Supplementary Table 3). S_obs_ varied significantly with respect to site (*p* < 2e-16), but not with respect to CO treatment (*p* = 0.242). In general, richness was highest for cultivated and flooded soils and lowest for sparsely vegetated or young volcanic soils and hot spring sites. Shannon diversity estimates ranged from 0.86 ± 0.70 (KKL-burned, no CO, 60°C) to 3.87 ± 0.29 (KRC, no CO, 60°C) (Figure 3B; Supplementary Table 3). The Shannon Index varied significantly with respect to site (*p* = 1.72e-12), but not with respect to CO treatment (*p* = 0.323). Shannon indices were highest for cultivated soil sites (KRC and KRC-2) and lowest for burned and unburned forest soils on Hawai’i (KKL and KKL-burned), for a young, vegetated volcanic soil (IG7), and for a sparsely vegetated volcanic soil from Iceland (KRF).

**Figure 4:**
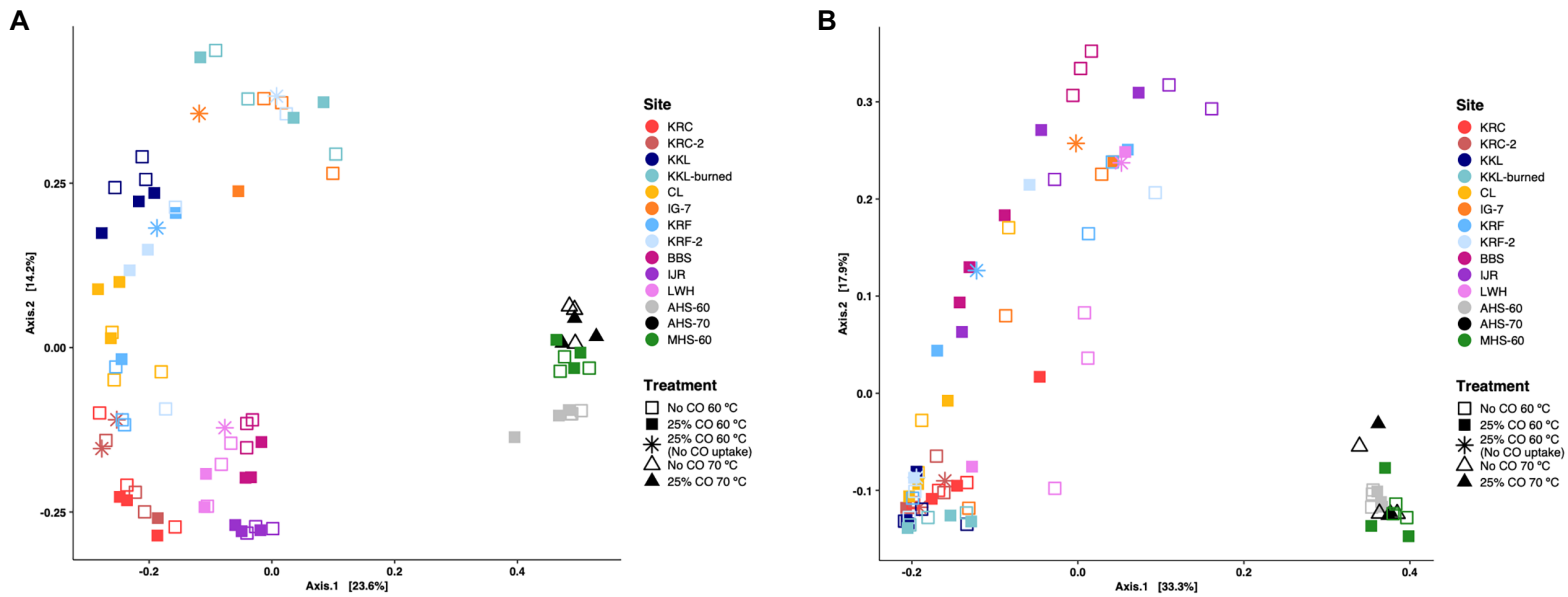
Principal coordinates analyses (PCoA) based on UniFrac distances for microbial communities derived from samples incubated under thermophilic conditions with or without 25% CO. **(A)** unweighted UniFrac metric; **(B)** weighted UniFrac metric. Samples are grouped by treatment and site; site abbreviations are described in the legend for Figure 1.

Both measures of beta diversity (weighted and unweighted UniFrac) showed that hot spring samples differed significantly in composition compared to all the other sites incubated at 60°C (Figure 4A). Ordinations using an unweighted UniFrac metric revealed significant differences among sites in community composition (*p* = 0.001) with exception of two communities obtained from the same location in two different years, KRC and KRC-2, which did not differ significantly (*p* = 0.444). However, CO addition did not significantly affect composition at any of the sites (*p* = 0.343; Figure 4A). In addition, the three sediment communities (BBS, IJR, LWH) were distinct from the various soil communities irrespective of their source or vegetation. Replicates within the samples for each site that exhibited no CO uptake were also distinct from the CO-oxidizing replicates at each site (Figure 4A). The dispersion of the samples for KRF-2 as determined by betadisper (vegan R package) was significantly different from the dispersions estimated for BBS, IJR, KRC, KRC-2, KKL, KKL-burned, LWH, and CL (*p* < 0.001; Supplementary Table 4).

Fewer significant differences among the communities were observed when using the weighted UniFrac metric (Figure 4B), however, consistent with unweighted UniFrac results, communities of KRC and KRC-2 did not differ significantly (*p* = 0.555). KRF microbial communities also did not differ significantly from those of KRF-2 (*p* = 0.244), IG-7 (*p* = 0.462), or CL (*p* = 0.10). KRF-2 microbial communities were not significantly different from those of CL (*p* = 0.208). In addition, the two flooded soil sites (BBS and IJR) did not differ (*p* = 0.150), nor did KKL and KKL-burned communities (*p* = 0.307). For each site, no significant differences in community composition were observed for incubation with exogenous 25% CO (*p* = 0.221). Pairwise comparisons of sites also showed no significant differences in dispersions (Supplementary Table 4). Similar outcomes were obtained when using only antherm-Firmicutes for beta diversity analysis; the sites were largely distinct with the notable exception that AHS and MHS clustered with other sediments in weighted UniFrac ordinations rather than separately as observed in whole community comparison (Supplementary Figures 1A,B).

### Candidate anaerobic thermophile ASV site occupancy

**Figure 5:**
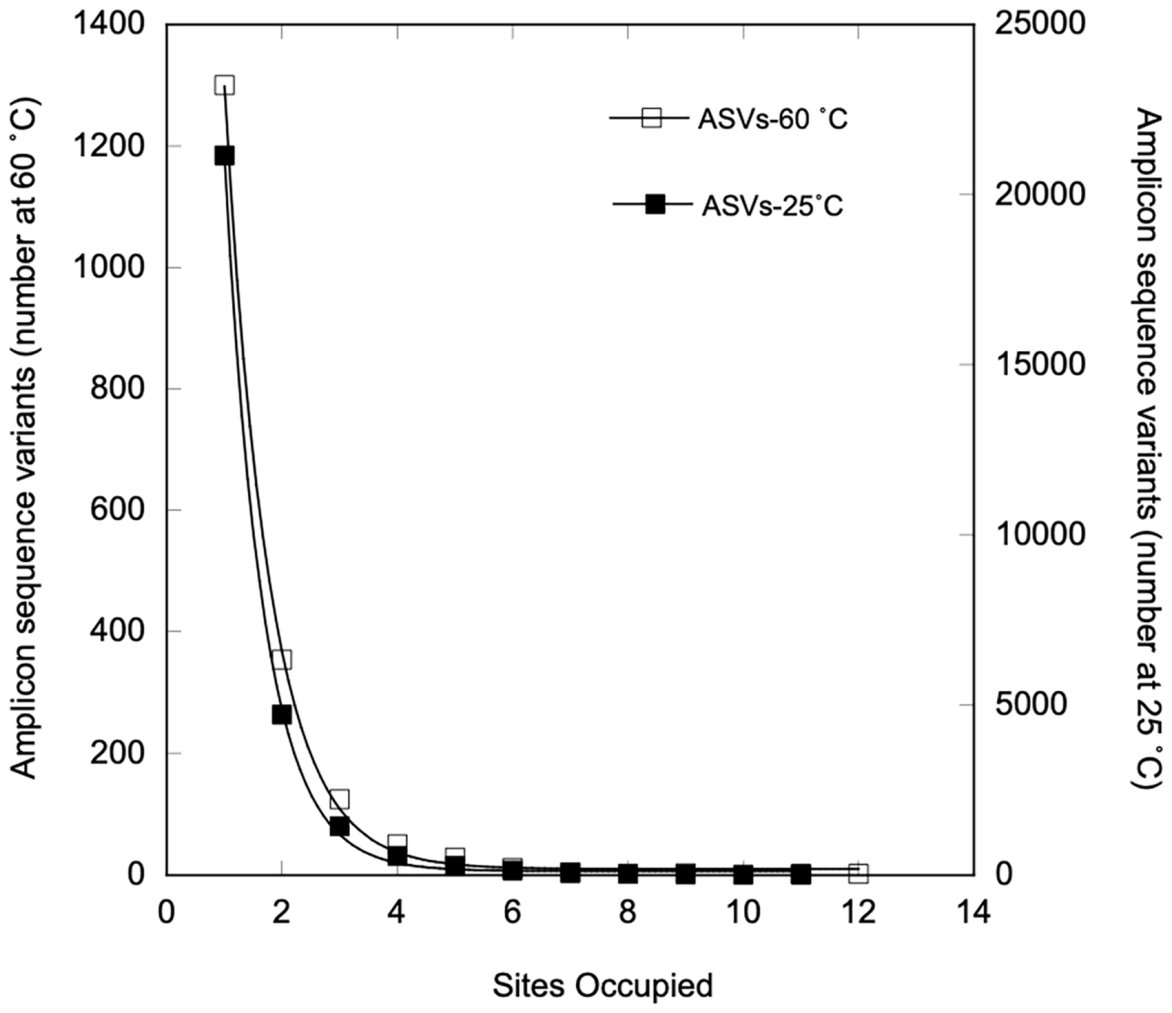
Numbers of sites occupied by individual ASVs for samples incubated at 25°C (open squares) or under thermophilic conditions (closed squares). The curve fit represents an exponential decay to a non-zero asymptote.

**Figure 6:**
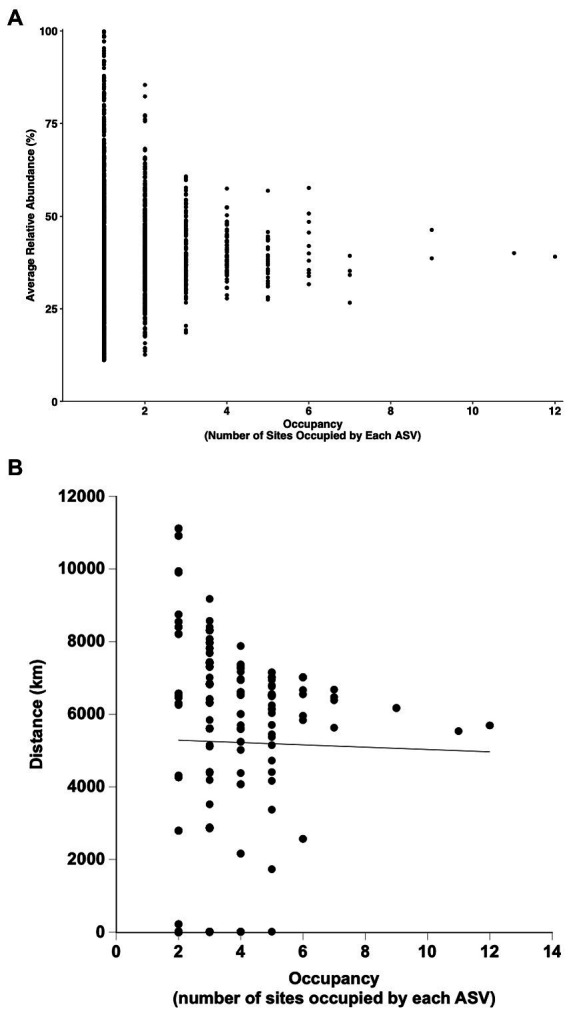
Occupancy patterns for ASVs derived from samples incubated under thermophilic conditions as a function of mean relative abundance **(A)** and mean distances among sites occupied **(B)**.

Two ASVs identified as *Anoxybacillus rupiensis* and *Parageobacillus* were broadly distributed, occupying 11 and 12 of 14 sites, respectively. Two other ASVs, which were identified as a *Thermoanaerobacterium* sp. and *Bacillus* sp., were also broadly distributed, occupying 9 out of 14 sites each. However, none of the ASVs occupied all of the sites, and 69.3% of 1873 ASVs occurred in only 1 site, while 18.8% were found in only 2 sites and just 5.29% occupied 4 or more sites (see Figure 5). The number of ASVs that occupied *n* sites decreased unimodally and exponentially with increasing site number to a predicted asymptotic limit of 9 ± 5 ASVs at 12 sites (r^2^ = 0.999; Figure 5); the extent of occupancy was not correlated with ASV relative abundance averaged over all of the sites (Figure 6A) or average distances among sites occupied by a given ASV (Figure 6B).

### Taxonomic composition and responses to CO for soils and sediments incubated at 25°C

Alpha-and BetaProteobacteria dominated soils as well as sediments with substantial contributions from Acidobacteria, Bacteroidota and Firmicutes. A more detailed summary has been provided in Supplementary Table 2. Although incubation with 25% CO did not significantly alter microbial community compositions at 25°C (Supplementary Table 2), some ASVs showed differential abundances based on a DESeq2 analysis. Examples include enrichments of *Geobacillus/Parageobacillus, Blastococcus,* and an unclassified Bacillaceae in cultivated soils and enrichments of *Clostridium, Acetobacterium,* unclassified Chitinophagaceae, *Anoxynatronum*, *Oscillibacter*, and *Desulfuromonas* in mesothermal hot spring soils (Supplementary Table 5).

### Beta diversity for mesothermal soils and sediments incubated at 25°C

At 25°C, ordinations using unweighted UniFrac revealed significant differences among the sites overall (*p* = 0.001), with pairwise comparisons indicating that each of the sites differed significantly from the others (Supplementary Figure 2A). However, significant differences were not observed among or between sites for the CO treatment (*p* = 0.182). The T_0_ samples clustered with the incubated samples for each site (Supplementary Figure 2A). In addition, the sediment sites were distinct from soil sites. Many of the sites also differed significantly with respect to dispersion among replicates (betadisper, *p* < 2.2e-16; Supplementary Table 6). Similarly, weighted UniFrac analyses revealed significant differences overall among the sites (*p* = 0.001; Supplementary Figure 2B). Additional pairwise comparisons indicated that each of the communities was distinct from the others. The low temperature hot spring sites were not only distinct from the other sites but were distinct from each other. Significant differences were observed for CO addition among the communities overall (*p* = 0.001) but pairwise comparisons indicated the differences were entirely attributable to site OY (Supplementary Figure 2B). In the weighted UniFrac analysis, sediment sites (BBS, IJR, and LWH) tended to cluster separately from soil sites, with the exception of some KRF-2 replicates (Supplementary Figure 2B). Many of the sites had distinct dispersions (betadisper, *p* = 1.367e-10; Supplementary Table 6).

### Mesophilic site occupancy

Regardless of sample type or location, most of the ASVs exhibited limited site occupancy (Figure 5). Of the 28,269 ASVs in the 25°C dataset, 74.8% occupied only one site, while just 3.5% occupied ≥ four sites. Only two ASVs (an unclassified Xanthobacteraceae and a member of the genus, *Xylophilus*) occurred in each of the 12 mesothermal sites. Four additional ASVs were present in 11 of 12 sites, including *Pseudarthrobacter, Devosia, Ramlibacter,* and an unclassified Burkholderiaceae. The occupancy of presumed mesophilic ASVs appeared to be unimodal with an exponential decrease to an asymptotic limit with increasing site number (*r*^2^ = 0.999, Figure 5). However, the predicted asymptotic limit for ASVs at 12 sites, 223 ± 115, greatly exceeded the observed limit of 1.

## Discussion

### Response to 60°C incubations: Thermophilic community composition and differentiation

The study presented here was designed to probe the composition and diversity of anaerobic thermophiles that emerged in unpasteurized mesothermal soils and sediments incubated *ex situ* at 60°C, with the addition of treatments containing CO at substrate levels (25%) to probe putative anaerobic thermophilic Ni-COX populations. In addition, a parallel analysis of two hot spring sites provided contrasts between communities that develop in response to pulsed short-term experimental heating versus long-term natural heating.

Although antherm-Firmicutes were not specifically enriched in this study as they were in the pasteurization-based study of Müller et al. (2014), they dominated (relative abundance) the taxa found in most of the soils and some of the sediments incubated at 60°C (Figure 1; Supplementary Table 2). In this context, they appear comparable to the aerobic thermophilic Firmicutes, which are dominant in experimentally heated oxic soils (Norris et al., 2002; Rahman et al., 2004; Marchant et al., 2008). However, several sites represented exceptions to the general trend. The reduced fraction of Firmicutes in a cultivated soil (KRC; 44–64%), an alpine lake sediment (LWH, 37–69%), and substantially lower fractions in hot spring sediments (AHS and MHS, 1–10%; Figure 1; Supplementary Table 2), suggest that antherm-Firmicutes spores might accumulate differentially *in situ* among habitats or respond differently to elevated temperatures during *ex situ* incubations.

Lower relative abundances of antherm-Firmicutes in KRC soils might be attributed to occasionally permissive *in situ* growth temperatures during the high desert summer of the Alvord Basin. Occasionally permissive temperature regimes could promote a greater diversity of thermophilic taxa that compete with Firmicutes compared to that in other soils; greater stresses on Firmicutes than occur in other soils; or perhaps both to some degree. Additional studies of managed soils and soils that experience elevated temperatures are needed to test this notion. Lower abundances of Firmicutes in LWH compared to the forested wetland (BBS) and rice paddy (IJR) sites might be attributed to differences in inputs of complex polymeric organics. In particular, inputs of plant cell wall polymers from macrophytes present in BBS and IJR but absent from LWH could select for greater relative abundances of cellulolytic/hydrolytic antherm-Firmicutes in the former. Nonetheless, it is noteworthy that CO addition substantially increased the relative abundance of antherm-Firmicutes in LWH (37–69%) while only limited to modest impacts were observed for other sites (Figure 1; Supplementary Table 2). More extensive comparative studies are needed to determine what trends exist in aquatic systems and to what variables, e.g., organic matter composition, they can be attributed.

AHS and MHS offer different insights, since they are continuously heated and thus represent habitats that select for active thermophilic populations that can form complex communities. Thus, like reports for other hot springs (Aanniz et al., 2015), AHS and MHS harbor numerous phylogenetically diverse thermophilic taxa (Figure 1; Supplementary Table 2), including antherm-Firmicutes. However, the relatively low abundance of the latter in AHS and MHS compared to the experimentally heated mesothermal soils and sediments suggests that they might not compete well in systems with consistently high temperatures. Negligible to low Firmicutes abundances reported by others for many hot springs, geothermally heated soils and hydrothermal vents support this proposition (Stott et al., 2008; Soo et al., 2009; Dick et al., 2013; Badhai et al., 2015; Gagliano et al., 2016; Merkel et al., 2016; Li and Ma, 2019; Noell et al., 2022). These results collectively indicate that spore formation by antherm-Firmicutes might promote essentially unrestricted dispersal but correlate with reduced competitive fitness in naturally heated systems where temperature is the major limiting variable (Filippidou et al., 2016).

Results of this study also reveal a broad distribution of candidate anaerobic thermophiles in phyla not known for resistant, readily dispersed life stages. In particular, thermophilic lineages of Acidobacteria (e.g., Thermoanaerobaculaceae [Losey et al., 2013] and Pyrinomonadaceae [Crowe et al., 2014]) were found in 11 of 14 sites, including hot springs (Supplementary Table 2). Similarly, several members of the anaerobic, thermophilic Alphaproteobacteria, *Acidicaldus* (Johnson et al., 2006), were found in multiple mesothermal soils and sediments though they were not detected in hot springs (Supplementary Table 2). Mechanisms that facilitate transport and survival of these taxa are unknown, since they do not produce spores and other resting stages have not been documented. However, it is clear that there is a need for transport models other than the aeolian deposition model used by Perfumo and Marchant (2010) to describe Firmicutes distribution.

Beta diversity analyses based on unweighted and weighted UniFrac distances revealed that distinct communities emerged among the various sites in response to anaerobic incubations at 60°C. T_0_ soil and sediment communities (Figures 4A,B) were also distinct, which suggests that the anaerobic thermophilic communities that developed during *ex situ* incubations might have reflected the impact of local environmental conditions to a greater extent than the nature of the source (s) of thermophilic taxa. Since atmospheric dispersal is not considered a major limiting factor for microbial distribution over ecological timescales (e.g., Herbold et al., 2014), environmental selection (or habitat filtering) and ecological drift likely account for the observed differences in composition among sites (e.g., Stegen and Hurlbert, 2011; Stegen et al., 2012, 2013).

**Figure 7:**
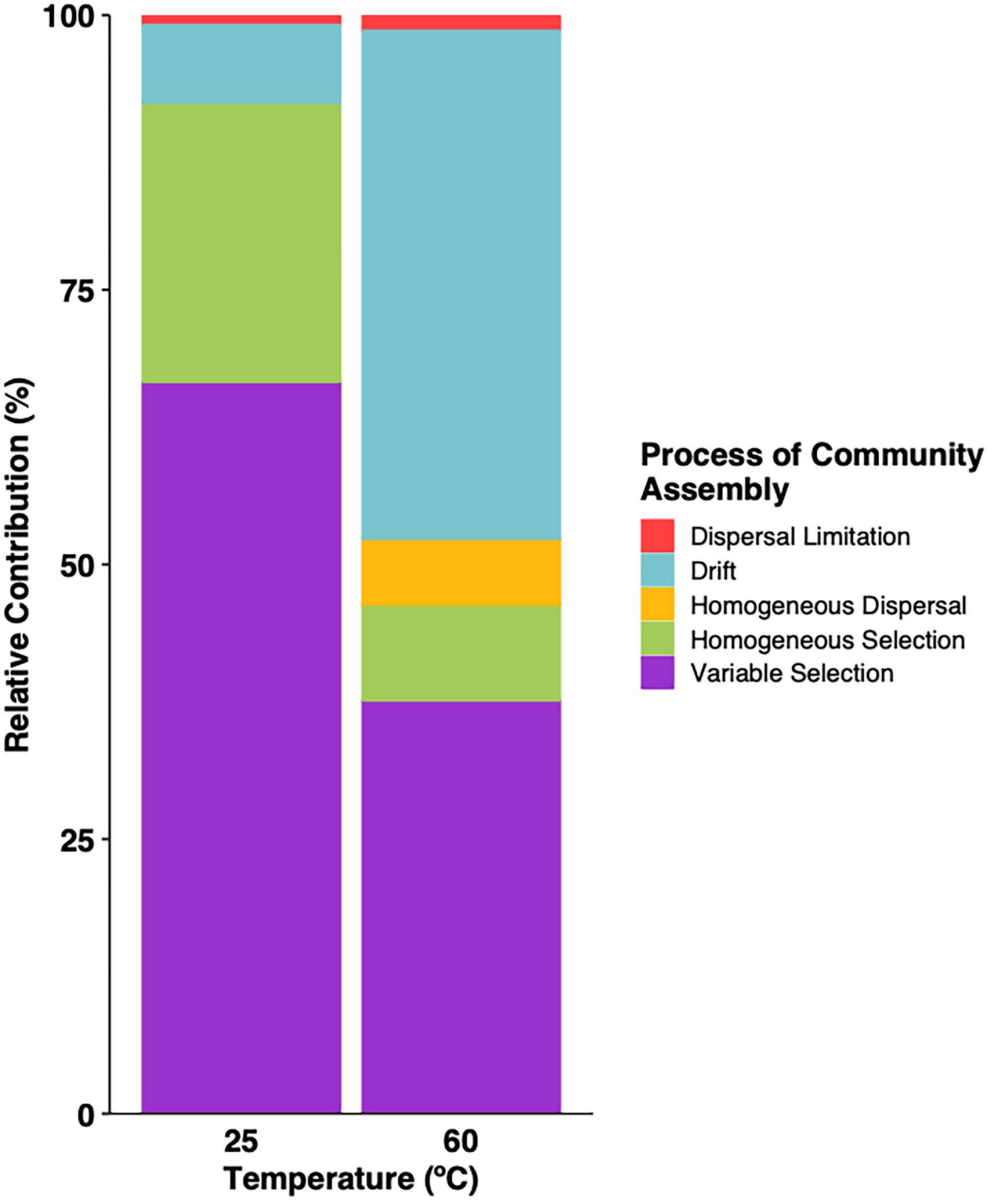
Relative contribution of ecological processes to community assembly for samples incubated under thermophilic conditions or at 25°C. Values were estimated using βMNTD and βNTI.

Several lines of evidence support this conclusion. First, sets of sites within close proximity that likely experience similar atmospheric inputs harbor distinct communities that correspond to clear differences in the nature of the sites. These include KKL and KKL-burned; CL, IG7 and OY; KRC, AHS and MHS (Figure 1). Environmental selection rather than dispersal limitation best explains results for these sites. Second, βMNTD and βNTI analyses (e.g., Stegen et al., 2012; Figure 7) indicate that environmental selection dominated the processes driving community assembly with dispersal contributing marginally in both the heated treatments and ambient incubations (Supplementary Table 7). Similar results have been obtained by others for mesothermal sub-surface communities (Stegen et al., 2013; Stegen et al., 2015), while a study of Tibetan lakes bacterioplankton indicated that core populations were structured by dispersal limitation and satellite populations were structured by selection (Yan et al., 2021). Outcomes in this study likely reflected the substantial environmental differentiation that exists among the various sites, including physical and chemical gradients that are greater than those in other studies.

A largely similar outcome was obtained when beta diversity analyses were restricted to the candidate antherm-Firmicutes ASVs at each of the sites (Supplementary Figures 1A,B). Communities comprised of antherm-Firmicutes were mostly distinct although the hot springs clustered with the soils and sediment sites in contrast to the outcomes obtained when using all ASVs, in which case the hot springs formed a separate, distinct cluster (Supplementary Figures 1A,B). This indicates that the continuously elevated temperatures at AHS and MHS did not select for populations of antherm-Firmicutes markedly different than those found in mesothermal soils and sediments, even though their relative abundances were low.

### Community responses to CO addition

CO was consumed anaerobically by soils and sediments from all sites incubated at both 25°C and 60°C as reported previously (DePoy et al., 2020; DePoy and King, 2022). The amount consumed (about 690 μmol per sample replicate) was sufficient to stimulate Ni-COX growth but imprecise mapping of 16S rRNA gene taxonomies and cell functions constrains inferences about the relationships between changes in ASV relative abundance and CO use. In addition, the potential for CO use by multiple Ni-COX taxa and the dynamics of other populations could limit observable changes in relative abundance and their interpretation. Nonetheless impacts of CO were detected for several ASVs by Deseq2 (Figure 2) and all sites harbored ASVs from genera with known thermophilic Ni-COX (e.g., *Parageobacillus*) or ASVs from genera that possess Ni-CODH (e.g., *Thermodesulfovibrio*). In particular, ASVs assigned to *Parageobacillus* and *Moorella* were widespread and in some cases substantially enriched in soils and sediments (Figure 1; Supplementary Table 2). While many *Parageobacillus* strains are aerobic, some thermophilic representatives respire nitrate (Nazina et al., 2001) and others, e.g., *P. thermoglucosidasius*, possess Ni-CODH and catalyze the anaerobic water-gas shift reaction (Brumm et al., 2015; Omae et al., 2019; Aliyu et al., 2021). The most widespread *Parageobacillus* ASV sequence in this study was identical to *P. thermoglucosidasius*, but additional effort, likely including metagenomic analyses, will be needed to assess which taxa occur where and under what conditions.

In contrast, all known *Moorella* isolates harbor Ni-CODH genes or consume CO anaerobically using Ni-CODH (Alves et al., 2013). In this study, a single relatively abundant *Moorella* ASV with a 16S rRNA sequence identical to that of the CO-oxidizing *M*. *thermoacetica* (formerly *Clostridium thermoaceticum* [Seifritz et al., 1999]) was widely distributed, occurring at 7 sites (soils, sediments and hot springs) with no evidence of dispersal limitation (Figure 1; Supplementary Table 2). *Parageobacillus* and *Moorella* thus collectively contribute to the potential for anaerobic thermophilic metabolism in soils and sediments as well as to anaerobic thermophilic CO cycling.

Several other putative Ni-COX taxa exhibited somewhat more restricted distributions. ASVs assigned to the non-sporing CO-oxidizing Firmicutes genus, *Carboxydothermus*, occurred only in sediments (BBS, IJR, LWH and AHS-60) where they responded to elevated temperatures and CO addition (Figure 1; Supplementary Table 2). This is consistent with the reported isolation of *Carboxydothermus* from hot springs and geothermally-heated sediments and enrichment of the genus after anaerobic incubation of sediments with CO (Slepova et al., 2009; Novikov et al., 2011; Yoneda et al., 2012, 2015). Results from this study along with the traits of existing isolates collectively suggest that *Carboxydothermus* might be able to disperse widely without spores or other known resistant stages and that it can be maintained in mesothermal sediments but perhaps cannot persist in mesothermal soils.

Four other Ni-COX genera, *Dictyoglomus* (Dictyoglomi), *Methanothermobacter* (Euryarchaeota), *Thermocrinis* (Aquificae), and *Thermodesulfovibrio* (Nitrospirae), were even more restricted, occurring only in the hot springs (Figure 1; Supplementary Table 2). Isolates for each of these genera have been obtained either from hot springs or heated engineered systems (Saiki et al., 1985; Wasserfallen et al., 2000; Eder and Huber, 2002; Sekiguchi et al., 2008; Caldwell et al., 2010; Kochetkova et al., 2011; Diender et al., 2015; Dodsworth et al., 2015; Brumm et al., 2016) but they have not been reported from experimentally heated mesothermal systems and were not observed in such systems in this study. They appear to represent a group of Ni-COX subject to strong environmental selection and whose dispersal might also be restricted.

Although multiple confirmed Ni-COX genera were observed in both AHS and MHS, they did not include taxa identified as Ni-COX by Brady et al. (2015), who used a ^13^C-stable isotope probing (SIP) approach coupled to 16S rRNA gene analyses to explore anaerobic CO oxidation in a set of Canadian hot springs with temperatures from 45 to 65°C. Brady et al. (2015) observed CO-oxidizing taxa including *Thermincola*, *Desulfotomaculum*, *Thermolithobacter,* and *Carboxydocella* and possibly *Caloramator* and *Thermus* as well.

In contrast, Omae et al. (2019) analyzed 16S rRNA gene sequences for a set of Japanese hot spring sediments that were not incubated with CO and identified putative Ni-COX including *Parageobacillus*, *Carboxydothermus, Carboxydocella*, and *Caldanaerobacter,* the first two of which were present in either AHS or MHS while the latter two were not. Reasons for different outcomes for these studies are not apparent but could include the different approaches (bulk community analyses versus SIP), different CO concentrations (25% versus 5–10% or 0%) or genuine differences in the three sets of hot spring communities. Additional comparative work with SIP and varied CO concentrations would provide valuable insights.

Although multiple ASVs were identified in this study as putative Ni-COX, including ASVs that were substantially enriched in the presence of CO, there was no statistically significant impact of CO addition on alpha diversity or beta diversity as measured by UniFrac metrics (Figures 4A,B). This suggests that while Ni-COX are routine members of anaerobic thermophilic communities, CO availability *in situ* might play only a limited role in determining their contribution to overall patterns of diversity. Since Ni-COX, like their aerobic counterparts, can typically use a variety of heterotrophic substrates, organic matter concentrations might be a more important factor. Future studies with organic substrates as treatments would provide a test of this notion.

### Occupancy analyses

Occupancy decreased unimodally with only two of 1873 candidate thermophile ASVs present at >10 sites (Figure 5). However, the sites from which these two ASVs were absent were geographically close to sites where they were present (Supplementary Tables 1, 2). ASV_36 (*P. glucosaidasius*) was absent from MHS-69 but present at a site less than 30 m away (MHS-60); ASV_36 was also absent from a soil in a burned forest (KKL-burned) but present at an unburned site about 1 km away (KKL). A comparable pattern was observed for ASV_1 (*A. rupiensis*), suggesting that these two ASVs are dispersed throughout the spatial region encompassed by the study with the characteristics of specific sites or stochasticity in responses to the *ex situ* incubations accounting for observed presence or absence.

Nonetheless, it is tempting to speculate that the 1298 ASVs that were restricted to individual sites provide evidence for dispersal limitation. However, dispersal limitation cannot be distinguished from environmental selection in this study, because habitat types were not specifically replicated across distance. In addition, 233 of the ASVs found at individual sites were spore-forming Firmicutes, which presumably are not limited by dispersal and for which localization at individual sites would best be attributed to other variables. Moreover, 261 of 276 ASVs that occupied two sites (excluding the hot springs) were separated by distances that exceeded 2000 km, and for ASVs occupying two or more sites, there was no statistically significant relationship between the number of sites occupied and the distances separating sites (Figure 6B; linear regression, *r*^2^ = 0.24, *p* = 0.17). In the context of this study, these observations are not consistent with dispersal limitation.

Occupancy results for the thermophiles in this study were also generally similar to outcomes for the 28269 ASVs obtained from the T_0_ mesothermal soils and sediments (Figure 5). This suggests that communities of both mesophiles and the thermophiles the emerge after heating mesothermal systems are structured by similar phenomena, primarily environmental selection. Nonetheless, occupancy patterns in this study differed in some respects from patterns in other studies of mesophiles (Chen et al., 2020; Lindh et al., 2017; Izabel-Shen et al., 2021; Mateus-Barros et al., 2021; Yan et al., 2021). In particular, occupancy in regional scale studies of bacterioplankton in the Baltic Sea (Lindh et al., 2017) and a set of Brazilian lakes (Mateus-Barros et al., 2021) exhibited bimodal patterns with an initial decline in occupancy as site numbers increased followed by an increase in the numbers of ASVs occupying the greatest site numbers. This distribution has been attributed to the presence of modest numbers of abundant core taxa with traits that permit growth under conditions that prevail at regional scales, along with large numbers of low abundance satellite taxa that grow under more limited conditions. The diversity of habitats in this study and their distribution across nearly 10,000 km mitigates against the likelihood of all but a few taxa with a capacity for growth under the widely different conditions that exist in the various soils and sediments. Further, the varied conditions mitigate against consistent growth of ASVs in the different sites where they emerge. Thus, there was no correlation between abundance and occupancy in this study (Figure 6A) in contrast to positive relationships observed by others for regional scale studies of systems with less dramatic physical and chemical gradients. Additional focused studies of sediments or soils at regional scales could help determine if populations of thermophiles that emerge after heating exhibit bimodal occupancy patterns similar to those of mesophiles or if other factors lead to unimodal patterns, e.g., stochasticity in responses to elevated temperatures.

## Conclusion

The ubiquity of aerobic thermophiles in mesothermal soils and sediments remains an enigma, in spite of evidence suggesting that aeolian deposition might account for at least regional scale dispersal (Perfumo and Marchant, 2010). Results reported here extend the enigma to anaerobic thermophiles - both sporing and non-sporing taxa as well as nickel-dependent anaerobic CO oxidizers - that must contend with the stresses of mesothermal conditions as well as molecular oxygen. Neither the sources of anaerobic thermophiles in terrestrial systems nor the mechanisms that promote their dispersal are understood, but outcomes of this study indicate that they experience environmental selection prior to or after experimental heating, which plays a major role in determining the composition of communities that emerge after heating. Occupancy analyses also indicated that a small number of anaerobic thermophiles are widely distributed while the majority of taxa are restricted to a limited number of often geographically distant sites. This pattern was similar to that observed for mesophiles assayed in parallel with the thermophiles, suggesting that the distribution and assembly of communities of both groups might be governed by the same principles. This in turn raises questions about the distribution and survival of other groups of extremophiles. For example, does the atmosphere serve as a medium for large-scale dispersal of acidophiles or alkaliphiles? Can members of either group persist in sub-optimal environments at low abundances and emerge when favorable conditions arise naturally or experimentally? What mechanisms or traits promote their dispersal and survival? In this context, extremophiles can serve as platforms to improve understanding of microbial biogeography.

## Data availability statement

The datasets presented in this study can be found in online repositories. The names of the repository/repositories and accession number(s) can be found in the article/Supplementary material.

## Author contributions

AD and GK contributed equally to experimental design, sample collection and processing, sample analysis, data analysis, and authorship. All authors contributed to the article and approved the submitted version.

## Funding

This research was funded in part by the United States National Science Foundation award EAR-15654499 and NASA award 15-EXO15_2-0147.

## Conflict of interest

The authors declare that the research was conducted in the absence of any commercial or financial relationships that could be construed as a potential conflict of interest.

## Publisher’s note

All claims expressed in this article are solely those of the authors and do not necessarily represent those of their affiliated organizations, or those of the publisher, the editors and the reviewers. Any product that may be evaluated in this article, or claim that may be made by its manufacturer, is not guaranteed or endorsed by the publisher.

## References

[ref1] Aanniz T. Ouadghiri M. Melloul M. Swings J. Elfahime E. Ibijbijen J.. (2015). Thermophilic bacteria in Moroccan hot springs, salt marshes and desert soils. Braz. J. Microbiol. 46, 443–453. doi: 10.1590/S1517-838246220140219, PMID: 26273259 PMC4507536

[ref2] Aliyu H. Kastner R. Mayer P. Neumann A. (2021). Carbon monoxide induced metabolic shift in the carboxydotrophic *Parageobacillus thermoglucosidasius* DSM 6285. Microorganisms 9:1090. doi: 10.3390/microorganisms9051090, PMID: 34069472 PMC8159138

[ref3] Alves J. I. van Gelder A. H. Alves M. M. Sousa D. Z. Plugge C. M. (2013). *Moorella stamsii* sp. nov., a new anaerobic thermophilic hydrogenogenic carboxydotroph isolated from digester sludge. Int. J. Syst. Evol. Microbiol. 63, 4072–4076. doi: 10.1099/ijs.0.050369-0, PMID: 23749275

[ref4] Badhai J. Ghosh T. S. Das S. K. (2015). Taxonomic and functional characteristics of microbial communities and their correlation with physicochemical properties of four geothermal springs in Odisha, India. Front. Microbiol. 6:1166. doi: 10.3389/fmicb.2015.01166 26579081 PMC4620158

[ref5] Barberán A. Henley J. Fierer N. Casamayor E. O. (2014). Structure, inter-annual recurrence, and global-scale connectivity of airborne microbial communities. Sci. Total Environ. 487, 187–195. doi: 10.1016/j.scitotenv.2014.04.030, PMID: 24784743

[ref6] Beule L. Karlovsky P. (2020). Improved normalization of species count data in ecology by scaling with ranked subsampling (SRS): application to microbial communities. PeerJ 8:e9593. doi: 10.7717/peerj.9593, PMID: 32832266 PMC7409812

[ref7] Brady A. L. Sharp C. E. Grasby S. E. Dunfield P. F. (2015). Anaerobic carboxydotrophic bacteria in geothermal springs identified using stable isotope probing. Front. Microbiol. 6:897. doi: 10.3389/fmicb.2015.00897, PMID: 26388850 PMC4555085

[ref8] Brumm P. J. Gowda K. Robb F. T. Mead D. A. (2016). The complete genome sequence of hyperthermophile *Dictyoglomus turgidum* DSM 6724 reveals a specialized carbohydrate fermenter. Front. Microbiol. 7:1979. doi: 10.3389/fmicb.2016.01979 28066333 PMC5167688

[ref9] Brumm P. Land M. L. Hauser L. J. Jeffries C. D. Chang Y.-J. Mead D. A. (2015). Complete genome sequence of *Geobacillus* strain Y4.1MC1, a novel CO-utilizing *Geobacillus thermoglucosidasius* strain isolated from bath hot spring in Yellowstone National Park. Bioenergy Res. 8, 1039–1045. doi: 10.1007/s12155-015-9585-2

[ref10] Caldwell S. L. Liu Y. Ferrera I. Beveridge T. Reysenbach A. L. (2010). *Thermocrinis minervae* sp. nov., a hydrogen-and sulfur-oxidizing, thermophilic member of the Aquificales from a costa Rican terrestrial hot spring. Int. J. Syst. Evol. Microbiol. 60, 338–343. doi: 10.1099/ijs.0.010496-0 19651724

[ref11] Callahan B. J. McMurdie P. J. Rosen M. J. Han A. W. Johnson A. J. A. Holmes S. P. (2016). DADA2: high-resolution sample inference from Illumina amplicon data. Nat. Methods 13, 581–583. doi: 10.1038/nmeth.3869, PMID: 27214047 PMC4927377

[ref12] Chen L. Liu S. Chen Q. Zhu G. Wu X. Wang J.. (2020). Dispersal limitation drives biogeographical patterns of anammox bacterial communities across the Yangtze River. Appl. Microbiol. Biotechnol. 104, 5535–5546. doi: 10.1007/s00253-020-10511-4, PMID: 32300854

[ref13] Cockell C. S. Cousins C. Wilkinson P. T. Olsson-Francis K. Rozitis B. (2014). Are thermophilic microorganisms active in cold environments? Int. J. Astrobiol. 14, 457–463. doi: 10.1017/S1473550414000433

[ref14] Crowe M. A. Power J. F. Morgan X. C. Dunfield P. F. Lagutin K. Rijpstra W. I. C.. (2014). *Pyrinomonas methylaliphatogenes* gen. nov., sp. nov., a novel group 4 thermophilic member of the phylum Acidobacteria from geothermal soils. Int. J. Syst. Evol. Microbiol. 64, 220–227. doi: 10.1099/ijs.0.055079-0, PMID: 24048862

[ref100] Dahl E. M. Neer E. Bowie K. R. Leung E. T. Karstens L. (2022). Microshades: An R package for improving color accessibility and organization of microbiome data. Microbiol. Resour. Announc. 11, 1–3., PMID: 36314910 10.1128/mra.00795-22 PMC9670991

[ref15] DePoy A. N. King G. M. (2022). Putative nickel-dependent anaerobic carbon monoxide uptake occurs commonly in soils and sediments at ambient temperature and might contribute to atmospheric and sub-atmospheric uptake during anoxic conditions. Front. Microbiol. 13:736189. doi: 10.3389/fmicb.2022.736189, PMID: 35401450 PMC8987735

[ref16] DePoy A. N. King G. M. Ohta H. (2020). Anaerobic carbon monoxide uptake by microbial communities in volcanic deposits at different stages of successional development on O-yama volcano, Miyake-jima, Japan. Microorganisms 9:12. doi: 10.3390/microorganisms9010012, PMID: 33375160 PMC7822213

[ref200] Dick G. J. Anantharaman K. Baker B. J. Li M. Reed D. C. Sheik C. S. (2013). The microbiology of deep-sea hydrothermal vent plumes: ecological and biogeographic linkages to seafloor and water column habitats. Front. Ext. Microbiol. 4, 1–16. doi: 10.3389/fmicb.2013.00124 PMC3659317 23720658

[ref17] Diender M. Stams A. J. M. Sousa D. Z. (2015). Pathways and bioenergetics of anaerobic carbon monoxide fermentation. Front. Microbiol. 6:1275. doi: 10.3389/fmicb.2015.01275, PMID: 26635746 PMC4652020

[ref18] Dixon P. (2003). A package of R functions for community ecology. J. Veg. Sci. 14, 927–930. doi: 10.1111/j.1654-1103.2003.tb02228.x

[ref19] Dodsworth J. A. Ong J. C. Williams A. J. Dohnalkova A. C. Hedlund B. P. (2015). *Thermocrinis jamiesonii* sp. nov., a thiosulfate-oxidizing, autotropic thermophile isolated from a geothermal spring. Int. J. Syst. Evol. Microbiol. 65, 4769–4775. doi: 10.1099/ijsem.0.000647 26419502

[ref20] Eder W. Huber R. (2002). New isolates and physiological properties of the Aquificales and description of *Thermocrinis albus* sp. nov. Extremophiles 6, 309–318. doi: 10.1007/s00792-001-0259-y, PMID: 12215816

[ref21] Filippidou S. Wunderlin T. Junier T. Jeanneret N. Dorador C. Molina V.. (2016). A combination of extreme environmental conditions favor the prevalence of endospore-forming Firmicutes. Front. Microbiol. 7:1707. doi: 10.3389/fmicb.2016.01707, PMID: 27857706 PMC5094177

[ref22] Fujimura R. Kim S.-W. Sato Y. Oshima K. Hattori M. Kamijo T.. (2016). Unique pioneer microbial communities exposed to volcanic sulfur dioxide. Sci. Rep. 6:19687. doi: 10.1038/srep19687 26791101 PMC4726209

[ref23] Fukuyama Y. Inoue M. Omae K. Yoshida T. Sako Y. (2020). Anaerobic and hydrogenogenic carbon monoxide-oxidizing prokaryotes: versatile microbial conversion of a toxic gas into an available energy. Adv. Appl. Microbiol. 110, 99–148. doi: 10.1016/bs.aambs.2019.12.001 32386607

[ref24] Gagliano A. L. Tagliavia M. D'Alessandro W. Franzetti A. Parello F. Quatrini P. (2016). So close, so different: geothermal flux shapes divergent soil microbial communities at neighbouring sites. Geobiology 14, 150–162. doi: 10.1111/gbi.12167, PMID: 26560641

[ref25] Godon J. J. Galès A. Latrille E. Ouichanpagdee P. Seyer J.-P. (2020). An “overlooked” habitat for thermophilic bacteria: the phyllosphere. BioDiscovery 23:e47033. doi: 10.3897/biodiscovery.23.e47033

[ref26] Guo Y. Fujimura R. Sato Y. Suda W. Kim S.-W. Oshima K.. (2014). Characterization of early microbial communities on volcanic deposits along a vegetation gradient on the island of Miyake, Japan. Microbes Environ. 29, 38–49. doi: 10.1264/jsme2.ME13142, PMID: 24463576 PMC4041228

[ref27] Herbold C. W. Lee C. K. McDonald I. R. Cary S. C. (2014). Evidence of global-scale aeolian dispersal and endemism in isolated geothermal microbial communities of Antarctica. Nat. Commun. 5:3875. doi: 10.1038/ncomms4875, PMID: 24846491

[ref28] Hernon F. Forbes C. Colleran E. (2006). Identification of mesophilic and thermophilic fermentative species in anaerobic granular sludge. Water Sci. Technol. 54, 19–24. doi: 10.2166/wst.2006.481, PMID: 16939079

[ref29] Izabel-Shen D. Hoger A. L. Jurgens K. (2021). Abundance-occupancy relationships along taxonomic ranks reveal a consistency of niche differentiation in marine bacterioplankton with distinct lifestyles. Front. Microbiol. 12:690712. doi: 10.3389/fmicb.2021.690712, PMID: 34262550 PMC8273345

[ref30] Johnson D. B. Stallwood B. Kimura S. Hallberg K. B. (2006). Isolation and characterization of *Acidicaldus organivorus*, gen. nov., spec. nov.: a novel sulfur-oxidizing, ferric iron-reducing thermo-acidophilic heterotrophic Proteobacterium. Arch. Microbiol. 185, 212–221. doi: 10.1007/s00203-006-0087-7, PMID: 16432746

[ref32] King G. M. Weber C. F. Nanba K. Sato Y. Ohta H. (2008). Atmospheric CO and hydrogen uptake and CO oxidizer phylogeny for Miyake-jima, Japan, volcanic deposits. Microbes Environ. 23, 299–305. doi: 10.1264/jsme2.ME08528, PMID: 21558722

[ref33] Kochetkova T. V. Rusanov I. I. Pimenov N. V. Kolganova T. V. Lebedinsky A. V. Bonch-Osmolovskaya E. A.. (2011). Anaerobic transformation of carbon monoxide by microbial communities of Kamchatka hot springs. Extremophiles 15, 319–325. doi: 10.1007/s00792-011-0362-7, PMID: 21387195

[ref34] Li L. Ma Z. (2019). Global microbiome diversity scaling in hot springs with dar (diversity-area relationship) profiles. Front. Microbiol. 10:118. doi: 10.3389/fmicb.2019.00118, PMID: 30853941 PMC6395440

[ref35] Lindh M. V. Sjostedt J. Ekstam B. Casini M. Lundin D. Hugerth L. W.. (2017). Metapopulation theory identifies biogeographical patterns among core and satellite marine bacteria scaling from tens to thousands of kilometers. Environ. Microbiol. 19, 1222–1236. doi: 10.1111/1462-2920.13650, PMID: 28028880

[ref36] Liu C. Cui Y. Li X. Yao M. (2021). microeco: an R package for data mining in microbial community ecology. FEMS Microbiol. Ecol. 97, 1–9. doi: 10.1093/femsec/fiaa255, PMID: 33332530

[ref37] Liu K. Linder C. R. Warnow T. (2011). RAxML and FastTree: comparing two methods for large-scale maximum likelihood phylogeny estimation. PLoS One 6:e27731. doi: 10.1371/journal.pone.0027731, PMID: 22132132 PMC3221724

[ref38] Logan N. A. Allan R. N. (2008). “Aerobic endospore-forming bacteria from Antarctic geothermal soils” in Microbiology of Extreme Soils. Soil Biology. eds. Dion P. Nautiyal C. S., vol. 13 (Berlin: Springer Verlag), 155–175.

[ref39] Losey N. A. Stevenson B. S. Busse H. J. Damste J. S. S. Rijpstra W. I. C. Rudd S.. (2013). *Thermoanaerobaculum aquaticum* gen. nov., sp. nov., the first cultivated member of Acidobacteria subdivision 23, isolated from a hot spring. Int. J. Syst. Evol. Microbiol. 63, 4149–4157. doi: 10.1099/ijs.0.051425-0, PMID: 23771620

[ref40] Louca S. (2022). The rates of global bacterial and archaeal dispersal. ISME J. 16, 159–167. doi: 10.1038/s41396-021-01069-8, PMID: 34282284 PMC8692594

[ref41] Love M. I. Huber W. Anders S. (2014). Moderated estimation of fold change and dispersion for RNA-seq data with DESeq2. Genome Biol. 15:550. doi: 10.1186/s13059-014-0550-8 25516281 PMC4302049

[ref42] Lozupone C. Knight R. (2005). UniFrac: a new phylogenetic method for comparing microbial communities. Appl. Environ. Microbiol. 71, 8228–8235. doi: 10.1128/AEM.71.12.8228-8235.2005, PMID: 16332807 PMC1317376

[ref43] Maki T. Lee K. C. Kawai K. Onishi K. Hong C. S. Kurosaki Y.. (2019). Aeolian dispersal of bacteria associated with desert dust and anthropogenic particles over continental and oceanic surfaces. J. Geophys. Res. Atmos. 124, 5579–5588. doi: 10.1029/2018JD029597

[ref400] Marchant R. Banat I. M. Rahman T. J. Berzano M. (2002). The frequency and characteristics of highly thermophilic bacteria in cool soil environments. Environ. Microbiol. 4, 595–602. 12366754 10.1046/j.1462-2920.2002.00344.x

[ref44] Marchant R. Franzetti A. Pavlostathis S. G. Tas D. O. Erdbrűgger I. Űnyayar A.. (2008). Thermophilic bacteria in cool temperate soils: are they metabolically active or continually added by global atmospheric transport? Appl. Microbiol. Biotechnol. 78, 841–852. doi: 10.1007/s00253-008-1372-y, PMID: 18256821

[ref300] Martinez Arbizu P. (2020). PairwiseAdonis: Pairwise multilevel comparison using adonis. R package version 0.4.

[ref45] Mateus-Barros E. de Melo M. L. Bagatini I. L. Caliman A. Sarmento H. (2021). Local and geographic factors shape the occupancy-frequency distribution of freshwater bacteria. Microb. Ecol. 81, 26–35. doi: 10.1007/s00248-020-01560-3, PMID: 32705311

[ref46] McMullan G. Christie J. M. Rahman T. J. Banat I. M. Ternan N. G. Marchant R. (2004). Habitat, applications and genomics of the aerobic, thermophilic genus *Geobacillus*. Biochem. Soc. Trans. 32, 214–217. doi: 10.1042/bst0320214 15046574

[ref47] McMurdie P. J. Holmes S. (2013). Phyloseq: an R package for reproducible interactive analysis and graphics of microbiome census data. PLoS One 8:e61217. doi: 10.1371/journal.pone.0061217, PMID: 23630581 PMC3632530

[ref48] Merkel A. Y. Pimenov N. V. Rusanov I. I. Slobodkin A. I. Slobodkina G. B. Tarnovetckii I. Y.. (2016). Microbial diversity and autotrophic activity in Kamchatka hot springs. Extremophiles 21, 307–317. doi: 10.1007/s00792-016-0903-1 28028613

[ref49] Müller A. L. de Rezende J. R. Hubert C. R. Kjeldsen K. U. Lagkouvardos I. Berry D.. (2014). Endospores of thermophilic bacteria as tracers of microbial dispersal by ocean currents. ISME J. 8, 1153–1165. doi: 10.1038/ismej.2013.225, PMID: 24351936 PMC4030223

[ref500] Nazina T. N. Tourova T. P. Poltaraus A. B. Novikova E. V. Grigoryan A. A. Ivanova A.. (2001). Taxonomic study of aerobic thermophilic bacilli: descriptions of *Geobacillus* subterraneus gen. nov., sp. nov. and *Geobacillus* uzenensis sp. nov. from petroleum reservoirs and transfer of *Bacillus stearothermophilus, Bacillus thermocatenulatus, Bacillus thermoleovorans, Bacillus kaustophilus, Bacillus thermoglucosidasius* and *Bacillus thermodenitrificans* to *Geobacillus* as the new combinations *G. stearothermophilus, G. thermocatenulatus, G. thermoleovorans, G. kaustophilus, G. thermoglucosidasius* and *G. thermodenitrificans*. Int. J. Syst. Evol. Microbiol. 51, 433–446. doi: 10.1099/00207713-51-2-433, PMID: 11321089

[ref50] Najar I. N. Thakur N. (2020). A systematic review of the genera *Geobacillus* and *Parageobacillus*: their evolution, current taxonomic status and major applications. Microbiology 166, 800–816. doi: 10.1099/mic.0.000945, PMID: 32744496

[ref51] Noell S. E. Baptista M. S. Smith E. McDonald I. R. Lee C. K. Stott M. B.. (2022). Unique geothermal chemistry shapes microbial communities on Mt.Erebus, Antarctica. Front. Microbiol. 13:836943. doi: 10.3389/fmicb.2022.836943, PMID: 35591982 PMC9111169

[ref52] Norris T. B. Wraith J. M. Castenholz R. W. McDermott T. R. (2002). Soil microbial community structure across a thermal gradient following a geothermal heating event. Appl. Environ. Microbiol. 68, 6300–6309. doi: 10.1128/AEM.68.12.6300-6309.2002, PMID: 12450855 PMC134386

[ref53] Novikov A. A. Sokolova T. G. Lebedinsky A. V. Kolganova T. V. Bonch-Osmolovskaya E. A. (2011). *Carboxydothermus islandicus* sp. nov., a thermophilic, hydrogenogenic, carboxydotrophic bacterium isolated from a hot spring. Int. J. Syst. Evol. Microbiol. 61, 2532–2537. doi: 10.1099/ijs.0.030288-0 21131500

[ref54] Omae K. Fukuyama Y. Yasuda H. Mise K. Yoshida T. Sako Y. (2019). Diversity and distribution of thermophilic hydrogenogenic carboxydotrophs revealed by microbial community analysis in sediments from multiple hydrothermal environments in Japan. Arch. Microbiol. 201, 969–982. doi: 10.1007/s00203-019-01661-9, PMID: 31030239 PMC6687684

[ref55] Perfumo A. Marchant R. (2010). Global transport of thermophilic bacteria in atmospheric dust. Environ. Microbiol. Rep. 2, 333–339. doi: 10.1111/j.1758-2229.2010.00143.x, PMID: 23766086

[ref56] Price M. N. Dehal P. S. Arkin A. P. (2010). FastTree 2 – approximately maximum-likelihood trees for large alignments. PLoS One 5:e9490. doi: 10.1371/journal.pone.0009490, PMID: 20224823 PMC2835736

[ref700] Quast C. Pruesse E. Yilmaz P. Gerken J. Schweer T. Yarza P.. (2013). The 1325 SILVA ribosomal RNA gene database project: improved data processing and 1326 web-based tools. Nucl. Acids Res. 41, D590–D596. doi: 10.1093/nar/gks1219, PMID: 23193283 PMC3531112

[ref57] Rahman T. J. Marchant R. Banat I. M. (2004). Distribution and molecular investigation of highly thermophilic bacteria associated with cool environments. Biochem. Soc. Trans. 32, 209–213. doi: 10.1042/bst0320209, PMID: 15046573

[ref58] Saiki T. Kobayashi Y. Kawagoe K. Beppu T. (1985). *Dictyoglomus thermophilum* gen. nov., sp. nov., a chemoorganotrophic, anaerobic, thermophilic bacterium. Int. J. Syst. Bact. 35, 253–259. doi: 10.1099/00207713-35-3-253

[ref59] Santana M. M. Gonzalez J. M. (2015). High temperature microbial activity in upper soil layers. FEMS Microbiol. Lett. 362, 1–4. doi: 10.1093/femsle/fnv182, PMID: 26424766

[ref60] Santl-Temkiv T. Amato P. Casamayor E. O. Lee P. K. H. Pointing S. B. (2022). Microbial ecology of the atmosphere. FEMS Microbiol. Rev. 46, 1–18. doi: 10.1093/femsre/fuac009, PMID: 35137064 PMC9249623

[ref61] Schmale D. G. 3rd Ross S. D. (2015). Highways in the sky: scales of atmospheric transport of plant pathogens. Annu. Rev. Phytopathol. 53, 591–611. doi: 10.1146/annurev-phyto-080614-115942, PMID: 26047561

[ref62] Seifritz C. Fröstl J. M. Drake H. L. Daniel S. L. (1999). Glycolate as a metabolic substrate for the acetogen *Moorella thermoacetica*. FEMS Microbiol. Lett. 170, 399–405. doi: 10.1111/j.1574-6968.1999.tb13400.x

[ref63] Sekiguchi Y. Muramatsu M. Imachi H. Narihiro T. Ohashi A. Harada H.. (2008). *Thermodesulfovibrio aggregans* sp. nov. and *Thermodesulfovibrio thiophilus* sp. nov., anaerobic, thermophilic, sulfate-reducing bacteria isolated from thermophilic methanogenic sludge, and emended description of the genus *Thermodesulfovibrio*. Int. J. Syst. Evol. Microbiol. 58, 2541–2548. doi: 10.1099/ijs.0.2008/000893-0, PMID: 18984690

[ref64] Slepova T. V. Sokolova T. G. Kolganova T. V. Tourova T. P. Bonch-Osmolovskaya E. A. (2009). *Carboxydothermus siderophilus* sp. nov., a *thermophilic*, hydrogenogenic, carboxydotrophic, dissimilatory Fe(III)-reducing bacterium from a Kamchatka hot spring. Int. J. Syst. Evol. Microbiol. 59, 213–217. doi: 10.1099/ijs.0.000620-0 19196756

[ref65] Smith D. J. Timonen H. J. Jaffe D. A. Griffin D. W. Birmele M. N. Perry K. D.. (2013). Intercontinental dispersal of bacteria and archaea by transpacific winds. Appl. Environ. Microbiol. 79, 1134–1139. doi: 10.1128/AEM.03029-12, PMID: 23220959 PMC3568602

[ref66] Soo R. M. Wood S. A. Grzymski J. J. McDonald I. R. Cary S. C. (2009). Microbial biodiversity of thermophilic communities in hot mineral soils of Tramway Ridge, Mount Erebus, Antarctica. Environ. Microbiol. 11, 715–728. doi: 10.1111/j.1462-2920.2009.01859.x, PMID: 19278453

[ref67] Stegen J. C. Hurlbert A. H. (2011). Inferring ecological processes from taxonomic, phylogenetic and functional trait beta-diversity. PLoS One 6:e20906. doi: 10.1371/journal.pone.0020906, PMID: 21698111 PMC3117851

[ref68] Stegen J. C. Lin X. Fredrickson J. K. Chen X. Kennedy D. W. Murray C. J.. (2013). Quantifying community assembly processes and identifying features that impose them. ISME J. 7, 2069–2079. doi: 10.1038/ismej.2013.93, PMID: 23739053 PMC3806266

[ref69] Stegen J. C. Lin X. Konopka A. E. Fredrickson J. K. (2012). Stochastic and deterministic assembly processes in subsurface microbial communities. ISME J. 6, 1653–1664. doi: 10.1038/ismej.2012.22, PMID: 22456445 PMC3498916

[ref600] Stegen J. C. Lin X. Fredrickson J. K. Konopka A. E. (2015). Estimating and mapping ecological processes influencing microbial community assembly. Front. Microbiol. 6:370. doi: 10.3389/fmicb.2015.00370, PMID: 25983725 PMC4416444

[ref70] Stott M. B. Saito J. A. Crowe M. A. Dunfield P. F. Hou S. Nakasone E.. (2008). Culture-independent characterization of a novel microbial community at a hydrothermal vent at Brothers volcano, Kermadec arc, New Zealand. J. Geophys. Res. Solid Earth 113. doi: 10.1029/2007JB005477

[ref71] Wasserfallen A. Nölling J. Pfister P. Reeve J. de Macario E. (2000). Phylogenetic analysis of 18 thermophilic *Methanobacterium* isolates supports the proposal to create a new genus, *Methanothermobacter* gen. nov., and to reclassify several isolates in three species, *Methanothermobacter thermautotrophicus* comb. nov., *Methanothermobacter wolfeii* comb. nov., and *Methanothermobacter marburgensis* sp. nov. Int. J. Syst. Evol. Microbiol. 50, 43–53. doi: 10.1099/00207713-50-1-43 10826786

[ref72] Wu X. L. Friedrich M. W. Conrad R. (2006). Diversity and ubiquity of thermophilic methanogenic archaea in temperate anoxic soils. Environ. Microbiol. 8, 394–404. doi: 10.1111/j.1462-2920.2005.00904.x, PMID: 16478446

[ref73] Yan Q. Deng J. Wang F. Liu Y. Liu K. (2021). Community assembly and co-occurrence patterns underlying the core and satellite bacterial sub-communities in the Tibetan Lakes. Front. Microbiol. 12:695465. doi: 10.3389/fmicb.2021.695465, PMID: 34745022 PMC8567192

[ref74] Yoneda Y. Kano S. I. Yoshida T. Ikeda E. Fukuyama Y. Omae K.. (2015). Detection of anaerobic carbon monoxide-oxidizing thermophiles in hydrothermal environments. FEMS Microbiol. Ecol. 91:fiv093. doi: 10.1093/femsec/fiv093 26223231

[ref75] Yoneda Y. Yoshida T. Kawaichi S. Daifuku T. Takabe K. Sako Y. (2012). *Carboxydothermus pertinax* sp. nov., a thermophilic, hydrogenogenic, Fe(III)-reducing, sulfur-reducing carboxydotrophic bacterium from an acidic hot spring. Int. J. Syst. Evol. Microbiol. 62, 1692–1697. doi: 10.1099/ijs.0.031583-0, PMID: 21908679

[ref800] Zeigler D. R. (2014). The *Geobacillus* paradox: why is a thermophilic bacterial genus so prevalent on a mesophilic planet?. Microbiology 160, 1–11. doi: 10.1099/mic.0.071696-0 24085838

