# Supplementary material for: Distribution and diversity of anaerobic thermophiles and putative anaerobic nickel-dependent carbon monoxide-oxidizing thermophiles in mesothermal soils and sediments
Source: Front Microbiol. 2023 Jan 9;13:1096186. doi: 10.3389/fmicb.2022.1096186 (PMC9868602; doi:10.3389/fmicb.2022.1096186)
Supplement: Supplementary file 1 [file Data_Sheet_1.zip › Supplementary Figures.docx]

Supplementary Figures

**Supplementary Figure 1.** Principal coordinates analyses (PCoA) based on UniFrac distances for microbial communities based on Firmicutes only derived from samples incubated under thermophilic conditions with or without 25% CO. (A), unweighted UniFrac metric; (B), weighted UniFrac metric.

Samples are grouped by treatment and site; site abbreviations are described in the legend for Fig. 1.

A

B

**Supplementary Figure 2.** Principal coordinates analyses (PCoA) based on UniFrac distances for samples incubated at 25 ºC with or without 25% CO. Samples are grouped by treatment and site using an unweighted UniFrac (A) or weighted UniFrac (B). Site abbreviations are described in the legend for Figure 1.

A

B
