# Supplementary material for: Distribution and diversity of anaerobic thermophiles and putative anaerobic nickel-dependent carbon monoxide-oxidizing thermophiles in mesothermal soils and sediments
Source: Front Microbiol. 2023 Jan 9;13:1096186. doi: 10.3389/fmicb.2022.1096186 (PMC9868602; doi:10.3389/fmicb.2022.1096186)
Supplement: Supplementary file 1 [file Data_Sheet_1.zip › Supplementary Table 3.docx]

| **Site** | **Treatment** | **S_obs_** | **Shannon** |
| --- | --- | --- | --- |
| KRC | No CO 60 ºC | 187.33 ± 11.9 | 3.87 ± 0.29 |
|  | 25% CO 60 °C | 222 ± 29.6 | 3.24 ± 0.87 |
| KRC-2 | No CO 60 ºC | 167.33 ± 25.7 | 3.00 ± 0.63 |
|  | 25% CO 60 ºC | 168 ± 9.24 | 3.08 ± 0.74 |
| CL | No CO 60 ºC | 100 ± 5.36 | 2.58 ± 0.14 |
|  | 25% CO 60 ºC | 78.7 ± 9.39 | 1.99 ± 0.14 |
| IG7 | No CO 60 ºC | 23.3 ± 1.20 | 1.07 ± 0.21 |
|  | 25% CO 60 ºC | 31.0 ± 8.00 | 1.14 ± 0.26 |
| OY | No CO 60 ºC | 32.0 (n = 1) | 2.06 (n = 1) |
| KKL | No CO 60 ºC | 25.7 ± 3.33 | 1.27 ± 0.24 |
|  | 25% CO 60 ºC | 24.0 ± 1.00 | 1.07 ± 0.25 |
| KKL-burned | No CO 60 ºC | 10.0 ± 1.00 | 0.86 ± 0.70 |
|  | 25% CO 60 ºC | 7.67 ± 2.03 | 0.90 ± 0.26 |
| KRF | No CO 60 ºC | 91.7 ± 7.31 | 1.52 ± 0.31 |
|  | 25% CO 60 ºC | 21.3 ± 1.86 | 1.02 ± 0.13 |
| KRF-2 | No CO 60 ºC | 42.7 ± 29.4 | 1.29 ± 0.62 |
|  | 25% CO 60 ºC | 32.0 ± 13.2 | 1.41 ± 0.25 |
| BBS | No CO 60 ºC | 87.3 ± 6.77 | 2.44 ± 0.12 |
|  | 25% CO 60 ºC | 100.7 ± 14.9 | 2.60 ± 0.24 |
| IJR | No CO 60 ºC | 204.3 ± 16.2 | 2.39 ± 0.44 |
|  | 25% CO 60 ºC | 125 ± 10.9 | 1.98 ± 0.35 |
| LWH | No CO 60 ºC | 91.0 ± 10.8 | 2.65 ± 0.31 |
|  | 25% CO 60 ºC | 74.0 ± 7.21 | 1.92 ± 0.63 |
| MHS-60 | No CO 60 ºC | 62.0 ± 3.46 | 3.01 ± 0.10 |
|  | 25% CO 60 °C | 58.3 ± 2.19 | 2.65 ± 0.33 |
| MHS-69 | 25% CO 60 °C | 24.0 (n = 1) | 2.09 (n = 1) |
| AHS-60 | No CO 60 ºC | 122 ± 8.89 | 3.47 ± 0.21 |
|  | 25% CO 60 ºC | 140 ± 8.89 | 3.77 ± 0.12 |
| AHS-70 | No CO 70 ºC | 62.3 ± 3.71 | 2.93 ± 0.27 |
|  | 25% CO 70 ºC | 66.7 ± 6.98 | 3.01 ± 0.10 |

**Supplementary Table 3.** Alpha diversity metrics (S_obs_ and Shannon) for samples incubated under thermophilic conditions with or without 25% CO; values are means of triplicates and standard errors unless otherwise indicated. Site abbreviations are described in the legend for Figure 1.
