# Supplementary material for: Distribution and diversity of anaerobic thermophiles and putative anaerobic nickel-dependent carbon monoxide-oxidizing thermophiles in mesothermal soils and sediments
Source: Front Microbiol. 2023 Jan 9;13:1096186. doi: 10.3389/fmicb.2022.1096186 (PMC9868602; doi:10.3389/fmicb.2022.1096186)
Supplement: Supplementary file 1 [file Data_Sheet_1.zip › Supplementary Table 7.docx]

**Supplementary Table 7.** Proportion of the community assembly attributed to ecological processes for samples at 60 ºC and 25 ºC. Values were estimated using βMNTD and βNTI.

| **Process of community assembly** | **Relative Contribution (%)** | |
| --- | --- | --- |
|  | **60 ºC** | **25 ºC** |
| Variable Selection | 37.6 | 66.6 |
| Homogeneous Selection | 8.73 | 25.4 |
| Dispersal Limitation | 1.37 | 0.79 |
| Homogeneous Dispersal | 5.99 | 0 |
| Drift | 46.3 | 7.14 |
